# Predicting and understanding comprehensive drug-drug interactions via semi-nonnegative matrix factorization

**DOI:** 10.1186/s12918-018-0532-7

**Published:** 2018-04-11

**Authors:** Hui Yu, Kui-Tao Mao, Jian-Yu Shi, Hua Huang, Zhi Chen, Kai Dong, Siu-Ming Yiu

**Affiliations:** 1 School of Computer Science, Northwestern Polytechnical University, Xi’an, China; 2 School of Life Sciences, Northwestern Polytechnical University, Xi’an, China; 3 School of Software and Microelectronics, Northwestern Polytechnical University, Xi’an, China; 4 Department of Critical Care Medicine, People’s Hospital of Jiangxi Province, Nan Chang, China; 5 Department of Computer Science, The University of Hong Kong, Hong Kong, China

**Keywords:** Drug-drug interaction, Nonnegative matrix factorization, Regression, Network community, Balance theory

## Abstract

**Background:**

Drug-drug interactions (DDIs) always cause unexpected and even adverse drug reactions. It is important to identify DDIs before drugs are used in the market. However, preclinical identification of DDIs requires much money and time. Computational approaches have exhibited their abilities to predict potential DDIs on a large scale by utilizing pre-market drug properties (e.g. chemical structure). Nevertheless, none of them can predict two comprehensive types of DDIs, including enhancive and degressive DDIs, which increases and decreases the behaviors of the interacting drugs respectively. There is a lack of systematic analysis on the structural relationship among known DDIs. Revealing such a relationship is very important, because it is able to help understand how DDIs occur. Both the prediction of comprehensive DDIs and the discovery of structural relationship among them play an important guidance when making a co-prescription.

**Results:**

In this work, treating a set of comprehensive DDIs as a signed network, we design a novel model (DDINMF) for the prediction of enhancive and degressive DDIs based on semi-nonnegative matrix factorization. Inspiringly, DDINMF achieves the conventional DDI prediction (AUROC = 0.872 and AUPR = 0.605) and the comprehensive DDI prediction (AUROC = 0.796 and AUPR = 0.579). Compared with two state-of-the-art approaches, DDINMF shows it superiority. Finally, representing DDIs as a binary network and a signed network respectively, an analysis based on NMF reveals crucial knowledge hidden among DDIs.

**Conclusions:**

Our approach is able to predict not only conventional binary DDIs but also comprehensive DDIs. More importantly, it reveals several key points about the DDI network: (1) both binary and signed networks show fairly clear clusters, in which both drug degree and the difference between positive degree and negative degree show significant distribution; (2) the drugs having large degrees tend to have a larger difference between positive degree and negative degree; (3) though the binary DDI network contains no information about enhancive and degressive DDIs at all, it implies some of their relationship in the comprehensive DDI matrix; (4) the occurrence of signs indicating enhancive and degressive DDIs is not random because the comprehensive DDI network is equipped with a structural balance.

## Background

When two or more drugs are taken together, they would unexpectedly influence each other in terms of pharmacokinetic or pharmacodynamical behavior [1]. This kind of influence is termed as Drug-Drug Interaction (DDI), which would cause adverse drug reactions (e.g. the reduction in efficacy or the increment on unexpected toxicity among the co-prescribed drugs). As the number of approved drugs increases, the number of unidentified DDIs is rapidly rising, such that more and more adverse effects among the drugs may occur. Unidentified DDIs would lead patients, who are treated with numerous medications, to be in the unsafe treatment of medication errors [2–5]. Understanding DDI is also the first step towards drug combination, which is one of promising strategies for multifactorial complex diseases [6]. Therefore, it is an urgent need to screen and analyze DDIs before clinical medications are administered. However, traditional experimental approaches for DDI identification (e.g. testing cytochrome P450 [7] or transporter-associated interactions [8]) face challenges, such as high cost, long duration, animal welfare considerations [9], the very limited number of participants and the great number of drug combinations under screening in clinical trials. As a result, only a few of DDIs could be identified during drug development (usually the clinical trial phase), and most of them are reported after the drugs are approved.

Computational approaches are promising to discover potential DDIs on a large scale, and they have gained many concerns from both academy and industry recently [10, 11]. Text-mining based computational approaches have been developed for detecting DDIs from different sources [9], including scientific literature [12, 13], electronic medical records [14], and the Adverse Event Reporting System of FDA (http://www.fda.gov). These approaches heavily rely on the clinical evidence in post-market, thus cannot provide alerts of potentially DDIs before clinical medications are administered. In contrast, machine learning-based computational approaches (e.g. naïve similarity-based approach [15], network recommendation-based [9], classification-based [16]) are able to provide such alerts by utilizing pre-marketed drug features or similarities [17], such as, chemical structures [15], targets [18], hierarchical classification codes [16] and side effects [9, 19]. Most of the existing machine learning-based approaches were designed for conventional binary prediction, which only indicates how likely a pair of drugs is a DDI. However, two interacting drugs may increase or decrease their own pharmaceutical behaviors or effects in vivo.

It is more important to know exactly whether the interaction increases or decreases the pharmaceutical behaviors of the drugs when making optimal patient care, establishing the dosage of a drug, designing prophylactic drug therapy, or finding the resistance to therapy with a drug [20]. As one of the important pharmaceutical indices, serum concentration reflects the amount of a drug in the pharmacokinetic circulation [21]. When interacting with other drugs, a drug would increase or decrease the level of its own and its partners’ serum concentration. For example, the serum concentration of Dofetilide (whose DrugBank Id is DB00204) decreases when it is taken with Dabrafenib (DB08912) together, whereas its serum concentration increases when taken with Dalfopristin (DB01764). For short, we name the first case of DDI as a degressive DDI and the second case of DDI as an enhancive DDI in the following texts.

To summarize, predicting approaches for comprehensive DDIs helps to uncover the underlying mechanism of how DDIs occur [19]. However, most of current approaches have been developed only for conventional binary DDIs, but not for enhancive and degressive DDIs. Furthermore, there is a lack of systematic analysis on the structural relationship hidden among known DDIs. Revealing such a structural relationship is very important, because it is able to help understand how DDIs occur. A promising solution of these two issues is able to guide medical doctors to make safe co-prescriptions.

In this paper, to address abovementioned issues, we firstly design a novel model (DDINMF) for DDI prediction based on Nonnegative Matrix Factorization (NMF). Representing DDIs as a binary network and a signed network respectively, we then make an attempt to reveal the structural relationship hidden among DDIs by NMF and social balance theory.

## Methods

### Dataset

We collected 2329 approved drugs from DrugBank [22] and selected 603 drugs, which have DDIs recorded in DrugBank. Among the set of drugs, we also removed those drugs without chemical structures or without the off-label side effects recorded in OFFSIDES [23], and kept the remaining 568 drugs having both of them. The final DDI dataset contains 21,351 DDIs, including 16,757 enhancive DDIs (E-DDI) and 4594 degressive DDIs (D-DDI). Each drug is represented as an 881-dimensional feature vector **f**_*str*_ based on PubChem structure descriptor and also a 9149- dimensional feature vector **f**_*se*_ according to the off-label side effects provided by OFFSIDES [23]. The second feature **f**_*se*_ was proposed firstly in [19]. These two vectors are binary, of which a value of 1 denotes the occurrence of a specific structure fragment in its chemical structure or the observation of a specific side effect in clinic, and 0 if this does not occur or is not observed. By organizing DDIs as a DDI graph, we also investigated the degrees of DDIs, enhancive DDIs and degressive DDIs respectively. The details of our DDI dataset are listed in Table 1.

**Table 1:** Details of comprehensive DDI network

Property	Value	Degree	Value	Degree of E-DDI	Value	Degree of D-DDI	Value
#Drug	568	Ave.	75.18	Ave.	59.00	Ave.	16.18
#DDI	21,351	Median	61.50	Median	45.00	Median	8.00
#E-DDI	16,757	Max.	296	Max.	230	Max.	206
#D-DDI	4594	Min.	1	Min.	0	Min.	0

*E-DDI*: enhancive DDIs, *D-DDI*: degressive DDIs

### Problem formulation

Without loss of generality, let **D** = {*d*_*i*_}, *i* = 1, 2, …, *m* be a set of *m* approved drugs. Their interactions can be organized as an *m* × *m* symmetric interaction matrix **A**_*m* × *m*_ = {*a*_*ij*_}, which can be regarded as the adjacent matrix of DDI network. For the conventional DDI, *a*_*ij*_ = 1 if *d*_*i*_ interacts with *d*_*j*_, and *a*_*ij*_ = 0 otherwise. For the comprehensive DDI, *a*_*ij*_ ∈ {−1, 0, +1}. In details, if *d*_*i*_ and *d*_*j*_ do not interact with each other, *a*_*ij*_ = 0. When there is an enhancive DDI or a degressive DDI between *d*_*i*_ and *d*_*j*_, *a*_*ij*_ =  + 1 or *a*_*ij*_ =  − 1 respectively. Obviously, as the special case of the comprehensive DDI matrix **A**, the conventional binary DDI matrix **A**_*b*_ can be obtained by **A**_*b*_ = *Binary*(**A**).

In addition, each drug *d*_*i*_ in **D** is represented as a *p*-dimensional feature vector **f**_*i*_ = [*f*_1_, *f*_2_, …, *f*_*k*_, …, *f*_*p*_], where *f*_*k*_ = 1 denotes the *k*-th specific structure fragment or observed side effect and *f*_*k*_ = 0 otherwise . All the drugs in **D** are sequentially organized as an *m* × *p* feature matrix **F**_*m* × *p*_.

**Fig. 1:**
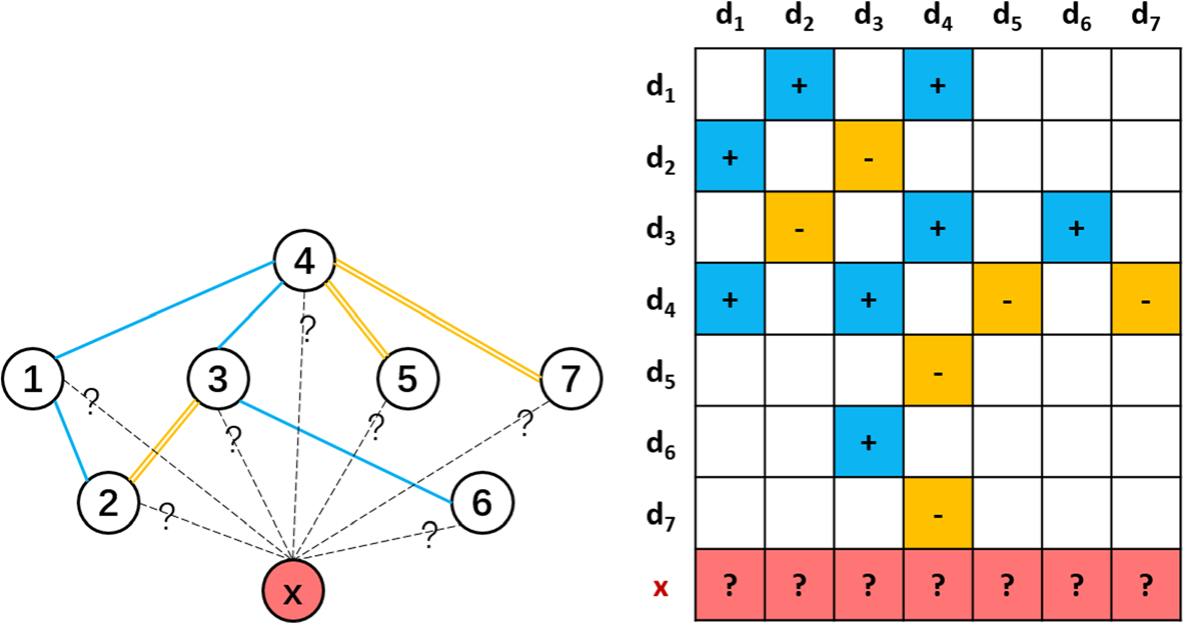
Illustration of predicting DDIs for a newly given drug. In the left panel, nodes in DDI network represent drugs. The hollow nodes are known drugs (numbered from 1 to 7) and the solid lines between them denote their interactions. Blue lines are enhancive interactions and yellow lines are degressive interactions. The node in red respectively is the newly given drug, tagged as **X**. In the right panel, the adjacent matrix is shown. The cells in it are filled with blue, yellow and red, accounting for the types of DDIs and drug pairs of interest respectively. All the pairwise entries among {*d*_1_,*d*_2_…,*d*_7_} are used to train the model, the entries in the red cells denote the testing entries. Our problem is to determine which known drugs could interact with the new drug **X** and what type these potential interactions are

To obtain a brief description, **a known drug** is referred to as a drug in **D** and **a new drug** is defined as the drug having no interaction with any drug in **D**. A screening scenario for new drugs is considered in this paper. Formally, the task is to predict how likely a newly given drug *d*_*x*_ interacts with one or more known drugs, and how likely a DDI of *d*_*x*_ is an enhancive DDI or a degressive DDI. Fig. 1 illustrates the task.

### Prediction model

**Fig. 2:**
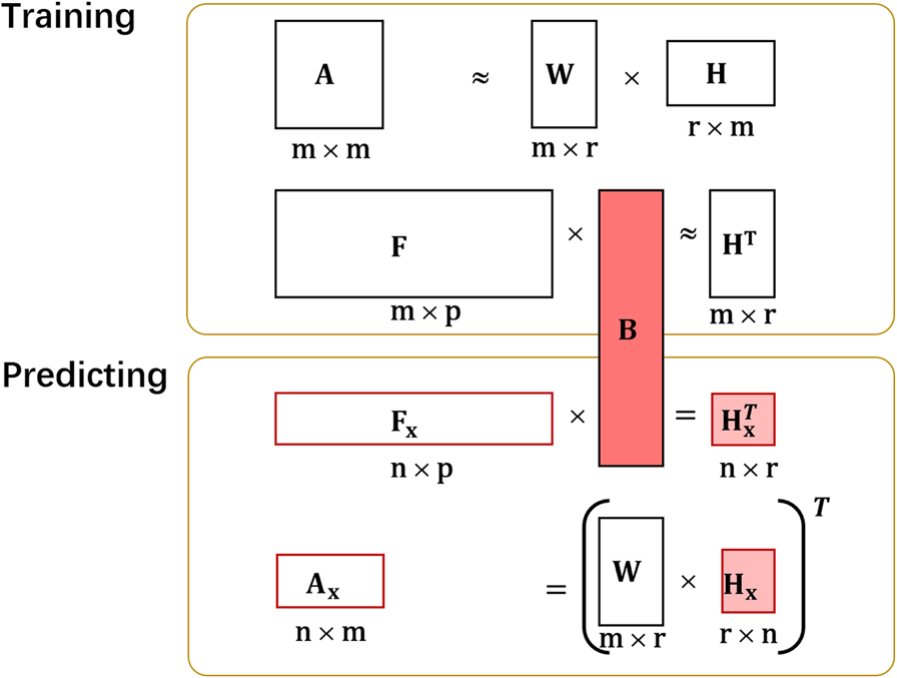
Overview of DDINMF. DDINMF contains a training phase and a predicting phase. (1) In its training phase, the adjacent matrix **A** is first decomposed into a basis (community) matrix and a latent (encoding) feature matrix by **A** ≈ **W** × **H**. Then the relationship between the input feature matrix **F** and the latent feature matrix **H** is modeled by a regression (**H**^*T*^) = **F** × **B**. (2) In the predicting phase, the learned regression coefficient **B** firstly maps the input feature matrix **F**_*x*_ of *n* newly given drugs into their latent feature matrix by ${\mathbf{H}}_x^T={\mathbf{F}}_x\times \mathbf{B}$. Then the mapped latent feature matrix of **F**_*x*_ is used to generate the predicted interactions between the new drugs and the known drugs by **A**_*x*_ = (**WH**_*x*_)^*T*^

Since the new drugs have no existing interaction with the drugs in **D** and are regarded as isolated nodes in the DDI network (Fig. 1), we cannot utilize their topological information to deduce their potential interactions. This is also the well-known cold start problem in recommendation system. Obviously, we need their additional properties (e.g. chemical structure fragments or side effects), which are called drug features in terms of machine learning. Once the additional features are obtained, we would build a supervised model to perform DDI prediction. The main idea is to build the relationship between the features of known drugs in **D** and their DDI network topology. We built the model (DDINMF) for predicting DDI based on nonnegative matrix factorization. It includes two phases (Fig. 2).

(1) In the training phase of DDINMF, the adjacent matrix **A**_*m* × *m*_ among *m* known drugs is first decomposed into two matrices **A**_*m* × *m*_ ≈ **W**_*m* × *r*_ × **H**_*r* × *m*_, where r is the dimension of the latent topological space. Denote **F**_*m* × *p*_ as the input feature matrix of those *m* known drugs (the training drugs). Then the relationship between **F**_*m* × *p*_ and **H**_*r* × *m*_ can be modeled by a regression (**H**^*T*^)_*m* × *r*_ = **F**_*m* × *p*_ × **B**_*p* × *r*_, where **B**_*p* × *r*_ is the regression coefficient matrix.

(2) In the predicting phase, the learned **B**_*p* × *r*_ firstly maps the *n* × *p* input feature matrix of *n* newly given drugs (denoted as **F**_*x*_) into the latent topological space by ${\mathbf{H}}_x^T={\mathbf{F}}_x\times \mathbf{B}$. Then the *n* × *r* mapped latent feature matrix **H**_*x*_ is used to generate the predicted interactions between the new drug and the known drugs by **A**_*x*_ = (**WH**_*x*_)^*T*^.

DDINMF applies the regular nonnegative matrix factorization (NMF) to decompose the DDI adjacent matrix when given a conventional binary adjacent matrix, and applies a variant of NMF, semi-NMF, when given a comprehensive adjacent matrix. A brief introduction of both NMF and semi-NMF can be found in the next section. The regression in DDINMF is solved by Partial Least Square Regression (PLSR) because there is a multi-collinearity between some columns of **F** or **H**. The model of multivariate PLSR can be solved by SIMPLS algorithm [24].

### Nonnegative matrix factorization and semi-nonnegative matrix factorization

Given an instance matrix **X**_*m* × *n*_ = [**x**_**1**_, **x**_**2**_, …, **x**_*n*_] ∈ ℝ^+^, Non-negative Matrix Factorization (NMF) aims to find a base matrix **W**_*m* × *r*_ = [**w**_*ir*_] ∈ ℝ^+^ and an encoding matrix **H**_*r* × *n*_ = [**h**_*ri*_] ∈ ℝ^+^, whose product can well approximate the original matrix **X**^+^ ≈ **W**^+^**H**^+^, where *r* < min(*m*, *n*). These approximating factors are typically obtained by solving the constrained least square minimization problem [25]:

1 $$ \underset{\begin{array}{l}W\ge 0\\ {}H\ge 0\end{array}}{\min }{\left\Vert \mathbf{X}-\mathbf{WH}\right\Vert}_F^2=\sum \limits_{i,j}{\left({\mathbf{x}}_{ij}-\sum \limits_{k=1}^r{\mathbf{w}}_{ik}{\mathbf{h}}_{ki}\right)}^2 $$

The algorithm minimizing the objective function is as follows:

2 $$ {\mathbf{w}}_{ik}\leftarrow {\mathbf{w}}_{ik}\frac{{\left({\mathbf{X}\mathbf{H}}^T\right)}_{ik}}{{\left({\mathbf{W}\mathbf{H}\mathbf{H}}^T\right)}_{ik}},\kern0.5em {\mathbf{h}}_{kj}\leftarrow {\mathbf{h}}_{kj}\frac{{\left({\mathbf{X}}^T\mathbf{W}\right)}_{kj}}{{\left({\mathbf{H}}^T{\mathbf{W}}^T\mathbf{W}\right)}_{kj}}\kern0.5em $$

The factor matrices **W** and **H** in the regular NMF are able to provide basic clustering. If we view **W** = [**w**_1_, **w**_2_, …, **w**_*r*_] as the cluster centroids, then **H** = [**h**_1_, **h**_2_, …, **h**_*n*_] can be viewed as the cluster indicators for each data-point. Especially, when **X**^+^ is a symmetric binary nonnegative matrix (e.g. the adjacent matrix of DDI **A**_*b*_), **W** denotes the communities in a network while **H** denotes the membership of instances to those communities.

Though there is a variant of NMF, symmetric NMF (SymNMF) [26], which looks more natural to the symmetric adjacent matrix of binary DDIs, it has disadvantages from a technical point of view. SymNMF has higher complexity than NMF, thus runs slower. Most importantly, it has a larger reconstructed error, especially a higher divergence of the diagonal elements in its reconstructed matrix comparing to all-zero diagonal of the original matrix. Therefore, we apply NMF but not SymNMF to decompose the binary adjacent matrix of DDI.

NMF has a strong constraint of **X** ∈ ℝ^+^. When the instance matrix has mixed signs (e.g. the adjacent matrix of comprehensive DDI **A** ), we consider Semi-NMF **X**^±^ ≈ **W**^±^**H**^+^, in which both $\mathbf{X}\in {\mathrm{R}}_{\pm}^{m\times n}$ and $\mathbf{W}\in {\mathbf{R}}_{\pm}^{m\times r}$ have mixed signs while only $\mathbf{H}\in {\mathbf{R}}_{+}^{r\times n}$ is restricted to comprise strictly nonnegative components [27]*.* Semi-NMF has the same form of cost/objective function as that of NMF, except for no constraint on **W**. But the algorithm minimizing the objective function is totally different:

3 $$ \mathbf{W}\leftarrow {\mathbf{XH}}^{\dagger },\kern0.5em \mathbf{H}\leftarrow \mathbf{H}\odot \sqrt{\frac{{\left({\mathbf{W}}^T\mathbf{X}\right)}^{\mathrm{pos}}+{\left[{\left({\mathbf{W}}^T\mathbf{W}\right)}^{\mathrm{neg}}\mathbf{H}\right]}_{ik}}{{\left({\mathbf{W}}^T\mathbf{X}\right)}^{\mathrm{neg}}+{\left[{\left({\mathbf{W}}^T\mathbf{W}\right)}^{\mathrm{pos}}\mathbf{H}\right]}_{ik}}}, $$

where **H**^†^ is the Moore–Penrose pseudo-inverse of **H**, **A**^pos^ is a matrix that has the negative elements of matrix **A** replaced with 0, and **A**^neg^ is the one that has the positive elements of **A** replaced with 0:

4 $$ \forall i,j.{\mathbf{A}}_{ij}^{\mathrm{pos}}=\frac{\mid {\mathbf{A}}_{ij}\mid +{\mathbf{A}}_{ij}}{2},{\mathbf{A}}_{ij}^{\mathrm{neg}}=\frac{\mid {\mathbf{A}}_{ij}\mid -{\mathbf{A}}_{ij}}{2}. $$

Though there are other methods of matrix factorization (e.g. Principal Component Analysis, PCA) that can be used to decompose the comprehensive adjacent matrix of DDIs, semi-NMF has the exclusive advantage that both its **W** and **H** show physical meanings, such as the ordinary degree, the difference of positive degree and negative degree, and the balance properties. See also Section *Results and Discussion*.

Finally, NMF and Semi-NMF are adopted to decompose the binary and the comprehensive adjacent matrices of DDIs in our predicting model respectively.

### Assessment

K-fold Cross-validation (K-CV) is the well-established approach to validate the power of generalization of algorithms in machine learning. To reflect the fact that new drugs have no interaction and to avoid over-optimistic prediction [17, 28–31], the K-CV scheme should be elaborately designed. For the given drugs having NO known interaction, the K-CV scheme tries to assess the task of predicting new potential interactions between them and those drugs having known interactions. The task corresponding to the K-CV scheme is useful when one tries to extend existing co-prescriptions by adding new drugs.

The generation of both training samples and testing samples is as follows. 1/K drugs are randomly removed out of all the given drugs in **D** as the testing drugs and the remaining drugs are taken as the training drugs. The drug pairs among the training drugs are selected as the training samples. The testing drugs are regarded as new drugs, and only the drug pairs between the testing drugs and the training drugs are selected as the testing samples, which are blind to the training (see also Fig. 1). Finally, the above procedures are repeated K times and the average of predicting performance in all rounds of CV is taken as the final performance. Usually, K = 10.

Two measures are usually adopted to assess the predicting performance, including the area under receiver operating characteristic curve (AUROC) and the area under precision-recall curve (AUPR) [32]. In the prediction of conventional binary DDIs, since positive and negative samples are interactions and non-interactions, both AUROC and AUPR can be calculated by comparing their predicted scores. Considering that the predicted scores of enhancive DDIs tend to be greater than ZERO and the predicted scores of degressive DDIs tend to be less than ZERO, we need to extend the calculation of AUROC and AUPR. In the prediction of comprehensive DDIs, to adopt the same way of calculating AUROC and AUPR, both enhancive and degressive DDIs first are labeled as positive samples while non-interactions are still labeled as negative samples. Then, the union of the predicted scores of enhancive DDIs and the MINUS of the predicted scores of degressive DDIs is compared with those scores of non-interactions by the same way as that in measuring conventional prediction.

## Results and Discussion

### Feature preprocessing

Because each drug was represented as an 881-dimensional feature vector **f**_*str*_ or as a 9149-dimensional feature vector **f**_*se*_, we reduced the high dimensions to accelerate the calculation. Though the value of the reduced dimension was unknown, we estimated it by performing ordinary PCA on the input feature matrix and counting the obtained PCs from the first one until the one, of which the entries having the values near to zeros. Then, using the number of PCs as the estimated dimension, we applied Kernel PCA [33] to the original input feature matrix and obtained the mapped feature matrix having significantly smaller dimensions. Gaussian function was adopted as the kernel in Kernel PCA with its variance equal to 4 for **f**_*str*_ and 16 for **f**_*se*_. Finally, the reduced dimensions of **f**_*str*_ and **f**_*se*_ were 487 and 557 respectively.

### Comparison with the state-of-the-art approaches

**Fig. 3:**
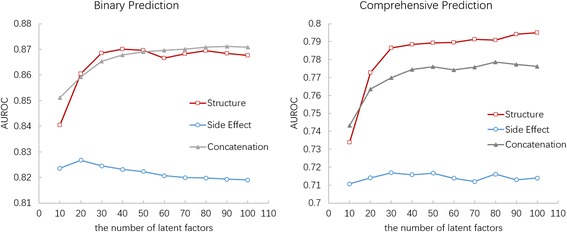
Illustration of determining the best value for the number of latent factors when given the structure feature and the side effect feature

Before running prediction, we assigned two parameters in our DDINMF, including the dimension of latent space (*r*) in matrix factorization and the number of latent factors (*k*) in PLSR as follows. We fixed the former with *rank*(**A**)/2 and tuned the latter from the list {10, 20, 30, 40, 50, 60, 70, 80, 90, 100} with the prediction measure of AUROC under 10-CV. Using the structure feature **f**_*str*_ and the side effect feature **f**_*se*_, we run NMF for binary DDI prediction and Semi-NMF for comprehensive DDI prediction respectively. When performing the conventional prediction of binary DDIs, we turned the entries equal to − 1 into + 1 in **A ** to form the binary DDI adjacent matrix **A**_*b*_ (see also Section *Problem Formulation*). The best values of the number of latent factors (*k* = 40 and *k* = 100 respectively) were picked up for two kinds of DDI prediction. In addition, we concatenated **f**_*str *_with **f**_*se*_ and run the prediction again (*k* = 100). The results show that **f**_*str*_ are significantly better than both **f**_*se*_ and their concatenation (Fig. 3). Therefore, only using the chemical structure feature **f**_*str*_, we performed DDI prediction in the following experiments.

First, we compared our approach with two state-of-the-art approaches, including Naïve similarity-based approach [15] and label propagation-based approach [9]. To obtain the robust predicting performance, 10-CV was repeated 50 times under different random seeds. Their final performance was reported by the average performance over 50 repetitions of CV (Table 2). The results show that DDINMF is significantly superior to them in terms of both AUROC and AUPR, and also indicate that the comprehensive prediction is more difficult than the conventional prediction.

**Table 2:** Comparison with state-of-the-art methods

	Binary Prediction		Comprehensive Prediction	
Method	AUROC	AUPR	AUROC	AUPR
Naïve Similarity [15]	0.779 ± 0.001	0.342 ± 0.002	0.641 ± 0.002	0.298 ± 0.004
Label Propagation [9]	0.776 ± 0.001	0.327 ± 0.002	0.635 ± 0.004	0.286 ± 0.006
DDINMF	0.872 ± 0.002	0.605 ± 0.006	0.796 ± 0.003	0.579 ± 0.003

We also compared NMF with SymNMF in the case of binary DDI prediction. We decomposed our binary DDI adjacent matrix with the dimension of latent space = *rank*(**A**)/2 by NMF and SymNMF respectively. NMF spends only 4.76 s on the binary adjacent matrix of DDIs while SymNMF spends 97.6 s using a computer equipped with Windows 10 (64-bits), Intel Core i7-4700MQ 2.40G and 16 GB RAM. On the other hand, the reconstructed error of NMF is only 26.1122 while that of SymNMF is up to 94.7374. The mean and standard derivation of the diagonal elements in the reconstructed matrix achieved by NMF are 0.3888 and 0.4003, whereas those achieved SymNMF are 1.4352 and 0.8303 respectively. In addition, the performance (AUROC = 0.870 and AUPR = 0.583) achieved by SymNMF is worse than those achieved by NMF (see also Table 2), especially on AUPR. In summary, NMF is better than SymNMF.

Similarly, we compared semi-NMF with PCA in the case of comprehensive DDI prediction. PCA achieves the prediction performance with AUROC = 0.780 and AUPR = 0.535. Obviously, semi-NMF with AUROC = 0.796 and AUPR = 0.579 outperforms PCA. More importantly, semi-NMF has the advantage that both its **W** and **H** show physical meanings, such as the ordinary degree, the difference of positive degree and negative degree, and the balance properties. See also next section.

Finally, considering the desirable performance of predicting conventional DDIs, we performed a novel prediction. In detail, we randomly collected 50 extra drugs from DrugBank and checked whether they interact with any of the original 568 drugs. According the predicted scores, the top-k drug pairs then were picked out and compared with the records in DrugBank. We counted the records matching the drug pairs of interest. For convenience, the number of such records over k is called hitting ratio. We checked the records from top-10 to top-100 with step 10 (Table 3). The validation demonstrates that our DDINMF is effective to predict DDIs for newly coming drugs.

**Table 3:** Novel Prediction

Top-k	10	20	30	40	50	60	70	80	90	100
Hitting ratio(%)	100	100	97	97	92	93	90	88	84	82

### Properties of DDI network

In this section, two questions are lifted up: (1) how the decomposed factor matrices **W** and **H** reflect the properties of DDI network; (2) whether the binary DDI matrix **A**_*b *_implies some relationship about enhancive and degressive DDIs in the comprehensive DDI matrix. Since NMF has the nature of clustering, the analysis based on it would give insights on the corresponding answers.

To describe briefly, we refer to a set of DDIs as a binary DDI network or a signed DDI network according to the type of DDI. For a drug, we also refer to the number of it DDIs, the number of its enhancive DDIs, and the number of its degressive DDIs as *Degree*, *Positive Degree*, and *Negative Degre*e respectively. In this context, we treated the rows of **W** as the community-derived features $\left\{{\mathbf{f}}_i^{\mathbf{W}}\right\}$ and the columns of **H** as the encoding features $\left\{{\mathbf{f}}_i^{\mathbf{H}}\right\}$ of drugs respectively. The former indicates how each drug contributes to comprise the communities, which account for all the columns of **W**, while the latter indicates how likely each drug belongs to those communities. Since their dimensions are high, we applied kernel PCA to them and used the first 3 principal components (PC) of the mapped instances to visualize them in a 3-d space.

**Fig. 4:**
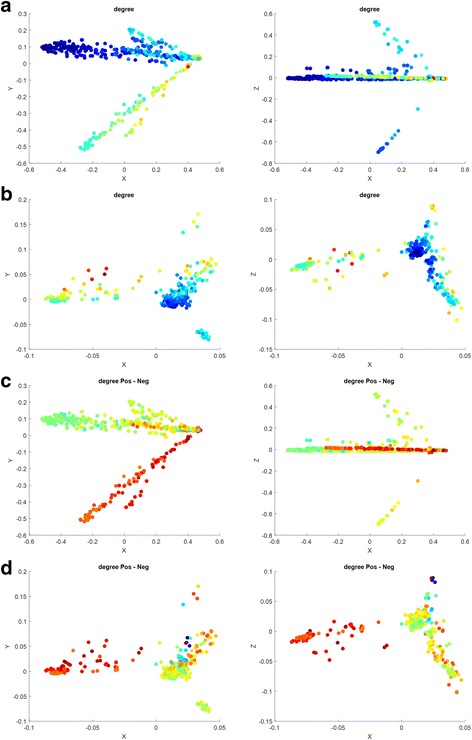
Illustration of the mapped space of binary DDI network. (**a**) Community-derived features rendered by drug degree; (**b**) encoding features rendered by drug degree; (**c**) community-derived features rendered by the difference between positive degree and negative degree; (**d**) encoding features rendered by the difference between positive degree and negative degree

We firstly tried to visualize the binary DDI network (Fig. 4). Each data-point in the two spaces is rendered by its drug degrees (Fig. 4a and c) and the difference between its positive degree and negative degree (Fig. 4b and d) respectively. In Fig. 4, Red means the largest degree or the degree difference, blue means the smallest value and other colors transiting from blue to red denote the middle values in an ascending order. Several interesting aspects can be found:

(1) As expected, the mapped spaces show significant clusters. Four significant branches are observed in the mapped space of community-derived features (Fig. 4a) while both two big clusters and one small cluster are found in the mapped space of encoding features (Fig. 4b). Degree shows a fairly clear distribution along those branches or in those clusters. For example, the Spearman Correlation between the degrees of the data points in the biggest branch (Fig. 4a) and their first mapped PC (denoted as X) is up to 0.9250.

(2) Surprisingly, the difference between Positive Degree and Negative Degree shows a significant distribution. Especially, both the branch X in Fig. 4c and the left cluster in Fig. 4d contain all the data points, of which their positive degrees are definitely greater than their negative degrees. In addition, the points having large degrees tend to have a large difference between Positive Degree and Negative Degree. The observation on binary DDIs surprisingly implies that the occurrence of signs indicating enhancive and degressive DDIs is not random.

**Fig. 5:**
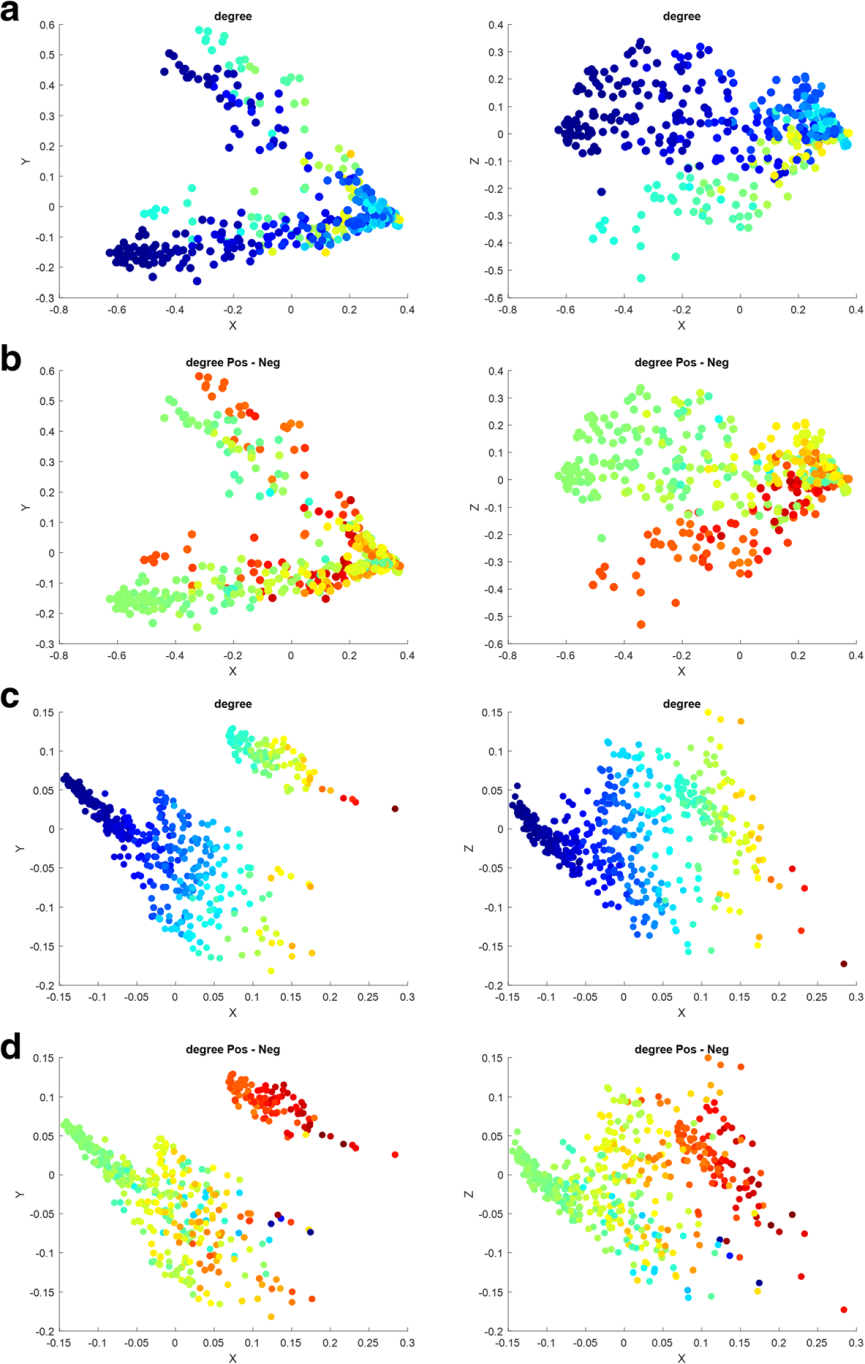
Illustration of the mapped space of comprehensive DDI network. (**a**) Community-derived features rendered by drug degree; (**b**) community-derived features rendered by the difference between positive degree and negative degree; (**c**) encoding features rendered by drug degree; (**d**) encoding features rendered by the difference between positive degree and negative degree

Similarly, we then tried to visualize the comprehensive DDI network (Fig. 5). The mapped space of community-derived features contains four sectors, which show a significant distribution of both degree and positive-negative degree difference in the plane of the first and the third PCs (denoted as X-Z). The mapped space of encoding features also shows that the positive degrees of data points are definitely greater than their negative degrees and that the points having large degrees tend to have a large difference between positive degree and negative degree. Differentially, the mapped space of encoding features presents that its first PC (denoted as X) and the degree are almost completely correlated with each other (Spearman Correlation = 0.9822). In general, the observation is consistent with that in Fig. 4.

In summary, one just keeps it in mind that the binary DDI network contains NO information about enhancive and degressive DDIs at all, but it shows a consistency with the comprehensive DDI network, especially about Degree, Positive Degree and Negative Degree. Therefore, the binary DDI network implies some relationship about enhancive and degressive DDIs in the comprehensive DDI matrix. In other words, the occurrence of signs indicating enhancive and degressive DDIs is not random.

To validate this point further, we checked the signed DDI network to see whether or not it is of a structural balanced network. The relationships in signed networks can be positive (“enhancive”, “like”, “friend”) or negative (“degressive”, “dislike”, “enemy”). Thus, there are four kinds of triangle relation patterns according to the type of DDI, including (positive, positive, positive), (positive, negative, negative), (negative, positive, positive) and (negative, negative, negative). The first two triangles are called the balanced pattern, the third is called as the unbalanced pattern and the last is named as the weakly balanced pattern. We counted four kinds of triangle patterns in the comprehensive (signed) DDI network and listed their numbers as follows: 374,215, 47,389, 46,478 and 7773. Obviously, ~ 90% triangles are balanced patterns. After checking the balance tendency in the mapped space of community-derived features, we found: (1) all the triangles (54,722) among the drugs having the first PC < 0 are purely balanced, and (2) the interactions of the drugs having the second PC < 0 are purely positive and negative in the two branches respectively. In addition, the small cluster in the mapped space of encoding features contains no weakly balanced triangles but 253,284 balanced triangles as well as 8597 imbalanced triangles. The results demonstrate that the comprehensive DDI network is equipped with a structural balance, such that the occurrence of enhancive and degressive DDIs is not random. The observation helps understand how DDIs gather together.

## Conclusions

Existing computational approaches are able to screen potential DDIs on a large scale before drugs are used in the medicine market. However, none of them can predict comprehensive DDIs, including enhancive and degressive DDIs, though it is important to know whether the interaction increases or decreases the behavior of the interacting drugs before making a co-prescription. Moreover, the important structural relationship hidden among DDIs still remains unknown.

To address the abovementioned issues, we have designed a novel approach DDINMF for comprehensive DDI prediction in different scenarios. Treating the set of DDI as a network, it provides a unified framework for predicting conventional binary DDIs as well as comprehensive DDIs. Experiments on the dataset demonstrate that DDINMF is significantly superior to two state-of-the-art approaches in the conventional binary DDI prediction and also shows an acceptable performance in the comprehensive DDI prediction. More importantly, DDINMF enables us to discover crucial knowledge hidden among DDIs, including degree distribution and tendency, as well as the implication of binary network to signed network, especially the structural balance of comprehensive DDI network.

There are still unknown but important factors among DDIs to be uncovered. In the future, we will focus on extracting and integrating more features of drug (e.g. drug targets and pathway) together to achieve improved DDI prediction, building an interpretable mapping between drug features and DDI, and digging out more structural patterns of comprehensive/signed DDI network and even their dynamic properties.

## Abbreviations

AUPR: The area under precision-recall curve
AUROC: The area under the receiver operating characteristic curve
CV: Cross-validation
DDI: Drug-drug interaction
NMF: Nonnegative matrix factorization
PCA: Principal Components Analysis
PLSR: Partial Least-Squares Regression
